# Electroconvulsive therapy and determination of cerebral dominance

**DOI:** 10.1186/1475-2832-3-14

**Published:** 2004-08-12

**Authors:** Milan Dragovic, Lindsay Allet, Aleksandar Janca

**Affiliations:** 1 Centre for Clinical Research in Neuropsychiatry, Graylands Hospital, Perth, Australia; 2 Inner City Mental Health Service, Royal Perth Hospital, Perth, Australia; 3 School of Psychiatry and Clinical Neurosciences, University of Western Australia, Perth, Australia

## Abstract

Electroconvulsive therapy (ECT) often results in a number of short- and long-time side effects including memory impairment for past and current events, which can last for several months after ECT treatment. It has been suggested that unilateral ECT (uECT) with electrodes placed over the non-dominant (typically right) hemisphere significantly reduces side effects, especially memory disturbances. It is important to note that cerebral dominance equates to speech dominance and avoiding this area of the brain also reduces speech dysfunction after ECT. Traditionally, the routine clinical determination of cerebral dominance has been through the assessment of hand, foot and eye dominance, which is an easy and inexpensive approach that, however, does not ensure accuracy. This review of literature on different methods and techniques for determination of cerebral dominance and provides evidence that functional transcranial Doppler sonography (fTCD) represents a valid and safe alternative to invasive techniques for identifying speech lateralisation. It can be concluded that fTCD, notwithstanding its costs, could be used as a standard procedure prior to uECT treatment to determine cerebral dominance, thereby further reducing cognitive side-effects of ECT and possibly making it more acceptable to both patients and clinicians.

## Introduction

Electroconvulsive therapy (ECT) is often regarded by the general public as a controversial procedure for the treatment of mental disorders. This is despite evidence of its safety and efficacy [1], and its benefit over anti-depressants in patients resistant to conventional medications and those with life threatening conditions such as catatonia and depressive stupor. The evidence suggests that in unipolar depression ECT has better efficacy when compared with older tricyclic antidepressants and monoamine oxidase inhibitors, as well as newer drugs such as paroxetine [2]. Notwithstanding the efficacy of ECT, its use is declining in some countries [3], while in a few others, including Italy – where ECT was first introduced in 1938 by Cerletti and Bini – it is prohibited. Aside from political reasons and public pressure, the declining trend in ECT use could be the result of the introduction of more effective antidepressants.

A further possible explanation for the reduction in ECT use may relate to the concern over adverse effects of the procedure. There are a number of short-term side effects including headache, nausea and, sometimes, brief confusion. However, the main side effect of concern is memory impairment for past events (retrograde amnesia) and for current events (anterograde amnesia) that can last for several months after a course of ECT treatment. Some of these side effects are substantially reduced by advances in safety and the introduction of controlled-current ECT machines. The utilisation of muscle relaxants, anaesthetics and resuscitation equipment, and electroencephalographic monitoring during the application of ECT are considered now considered routine. In addition, ECT guidelines issued by the UK National Institute for Clinical Excellence [4] restrict the use of ECT only to patients with severe symptoms to which "an adequate trial of other treatment options has proven ineffective" (p. 5). The risk associated with ECT has also been reduced with the introduction of refined ECT procedures, such as "maintenance ECT" or "unilateral ECT" (uECT) [5].

It has been suggested that unilateral treatment significantly reduces side effects, especially memory disturbances [6,7]. Despite the well-documented efficacy of unilateral over bilateral ECT, current practice still favours bilateral treatments [8,9]. Unilateral treatment, for the majority of patients, entails that electrodes are placed over the non-dominant, right hemisphere. Given that memory impairment could be reduced by unilateral electrode placement and the fact that placement of electrodes to the dominant hemisphere may cause a greater disturbance in memory compared to non-dominant uECT, determination of cerebral dominance appears to be critical [10]. It is important to note that cerebral dominance here equates to speech dominance, including a lateralised capacity of the cortex to be the locus of language-specific memory traces [11]. Avoiding the stimulation of the speech area will therefore reduce speech dysfunction after ECT. Traditionally, the routine clinical determination of cerebral dominance has been through the assessment of hand, foot and eye dominance. It certainly is an easy and inexpensive approach, but it does not ensure accuracy.

## Unilateral ECT and cerebral dominance

The practice of determining cerebral dominance from handedness appears to mirror Broca's view that a person's handedness is opposite to hemispheric language specialisation. This, however, is incorrect, since there is no "mirror-image" cortical language organisation in left-handers. Several attempts to improve cerebral dominance assessment by introducing additional clues such as handwriting posture (i.e. inverted or hooked style versus non-inverted) and familial sinistrality have not substantially improved the prediction as to determination of cerebral dominance [12,13]. For example, the use of hand writing posture to determine speech dominance has been shown to be completely invalid [14-16].

A great majority of left-handers have also an ipsilateral functional specialisation for language (i.e. left hemispheric, as in the majority of right-handers). Although right-handers are more clearly lateralised than left-handers in this regard, a certain proportion of right-handers have language localised in the right-hemisphere. This has been confirmed by various techniques, ranging from the old and invasive procedures such as the intracarotid sodium amytal test and ECT, to the new and more sophisticated techniques such as functional magnetic resonance imaging (fMRI) and functional transcranial Doppler sonography (fTCD). The pooling of empirical data from a number of studies [17-27] which used both old and new, non-invasive techniques to determine cerebral dominance for language is shown in Table 1.

**Table 1:** Percentages of right- and left-handers with speech localised predominantly in the left, right hemisphere, or bilaterally, according to different studies and techniques

Study	Right-handers			Left-handers		
	
	Left	Bilateral	Right	Left	Bilateral	Right
Milner, 1975*	96	0	4	70	15	15
Rossi & Rosadini, 1967*	99	1	0	40	10	50
Pratt & Warrington, 1972^#^	99	0	1	-	-	-
Warrington & Pratt, 1973^#^	-	-	-	70	7	23
Geffen et al., 1978^#^	92	0	8	67	0	33
Geffen & Traub, 1979^‡^	84	9	7	61	15	24
Springer et al. 1999^&^	94	6	0	-	-	-
Pujol et al. 1999^&^	96	4	0	76	14	10
Szaflarski et al. 2002^&^	-	-	-	78	14	8
Hund-Georgiadis et al. 2002^&^	94	0	6	47	12	41
Knecht et al. 2000^†^	-	-	4	-	-	27

* intracarotid sodium amytal test

^# ^ECT

^‡ ^dichotic listening test

^&^fMRI

^† ^fTCD

From Table 1 one can see that if the hemisphere for uECT treatment were solely ascertained from handedness assessment, then a small proportion of right-handers and a much larger proportion of left-handers would have treatment administered to the dominant hemisphere. One could also see from it that about 3% of right-handers and 25% of left-handers have speech localised in the right hemisphere. This represents the error rate percentage in both groups if uECT was administered to all patients on the right side of the cranium. However, the overall error rate is lower since the incidence of left-handedness is low, and is likely to be in the range of 6.4% to 12.5% [28]. A strict application of the "mirror-image" cortical organisation (i.e. considering left-handers as right-hemisphere dominant and therefore performing left-sided uECT) is even more destructive, illogical, and would increase the error rate. For example, in the survey of the use of ECT by psychiatrists in New Zealand [9], 20% of respondents reported using uECT depending on handedness. Adverse effects caused by determining speech dominance on the basis of handedness would be lower if right-sided ECT was always administered, thus making handedness assessment unnecessary. Given the additional risk of uECT treatment on the dominant hemisphere, which is even more disruptive than bilateral ECT [29], correct identification of cerebral dominance appears to be crucial. The importance of identification of cerebral dominance prior to electrode placement has been highlighted by a number of authors [6,10,28], but routine ECT practice has remained unchanged.

## From intracarotid injection to transcranial sonography

Until recently, an accurate determination of speech dominance prior to a course of ECT treatment was possible only through invasive procedures such as intracarotid sodium amytal test [30], also known as the Wada tests, and through the administration of ECT itself – the ECT Test [10]. Lateralisation of language capacity using the Wada test is based on the temporary anaesthesia of one half of the brain. The subject in the study receives sodium amytal – a short-acting anaesthetic – into (usually) the left carotid artery. This causes the left hemisphere to be temporarily rendered dysfunctional. As a result, if this were the patient's dominant hemisphere, the subject's language capacity – primarily speech production – is affected. Conversely, injecting sodium amytal into the right carotid artery leaves this language capacity intact. By using this technique it is possible to identify precisely which hemisphere hosts language, which is considered necessary for patients who are to go through neurosurgical procedures. Although accurate, the use of Wada procedure in a normal healthy population is generally considered as unsuitable. Using ECT for the determination of cerebral dominance is, as mentioned previously, associated with adverse effects and therefore may not be entirely appropriate, although Weiner [31] suggests giving left and right side ECT alternately followed by the administration of a simple verbal performance test and then continuing treatment with the side associated with the better result.

The advent of sophisticated and non-invasive technologies during the 1980s and 1990s has enabled a non-invasive approach to the assessment of speech dominance. One of the most elegant, mobile, and cost effective methods for determining cerebral dominance for speech is functional transcranial Doppler sonography (fTCD). fTCD is increasingly used in both clinical and research settings and is a new and robust technique based on the same principles as fMRI. Subjects in studies using this method are asked to generate as many possible words within 5-second periods after a letter presented on the computer screen cues for word generation. Basically, fTCD measures cerebral blood flow velocity which corresponds to brain activity. The physical foundation for this technique is quite old and is based on the work of the Austrian mathematician and physicist, Christian Doppler (1803–1853), who discovered that the change in pitch results from a shift in the frequency of the sound waves. This means that the speed of a physical object (i.e. blood) can be estimated by measuring the rate of change of pitch. To complete the fTCD procedure, the additional sound produced through the arteries is required.

Recently, it has been argued that fTCD can reliably replace the Wada procedure in patients undergoing brain surgery [32]. The validity of fTCD has been established by comparing fTCD with the Wada test, which is considered as the ultimate (gold standard) test of cerebral lateralisation for speech. Several independent studies [33-35] have found highly significant correlations between these two methods. A high agreement between fTCD and fMRI has also been identified [36] for the assessment of cerebral speech lateralisation.

## Conclusion

This review of the literature on ECT and cerebral dominance provides evidence that fTCD represents a valid and safe alternative to invasive techniques for identifying speech lateralisation. It seems therefore, that fTCD, notwithstanding costs, could be used as a standard procedure prior to uECT treatment to determine cerebral dominance, thereby further reducing cognitive side-effects of ECT and possibly making it more acceptable to both patients and clinicians.

## Competing interests

none declared.
